# A Low-Power, Auto-DC-Suppressed Photoplethysmography Readout System with Differential Current Mirrors and Wide Common-Mode Input Range Successive Approximation Register Analog-to-Digital Converter

**DOI:** 10.3390/mi16040398

**Published:** 2025-03-29

**Authors:** Chanyoung Son, Seok-Tae Koh, Hyuntak Jeon

**Affiliations:** 1 School of Semiconductor Engineering, Chungbuk National University (CBNU), Cheongju 28644, Republic of Korea; cyson@cbnu.ac.kr; 2 School of Electrical Engineering, Chungbuk National University (CBNU), Cheongju 28644, Republic of Korea

**Keywords:** PPG (photoplethysmography), readout ICs (ROICs), differential current mirror (DCM), successive approximation register (SAR), analog-to-digital converter (ADC)

## Abstract

This paper presents a low-power photoplethysmography (PPG) readout system designed for wearable health monitoring. The system employs a differential current mirror (DCM) to convert single-ended PPG currents into differential voltages, inherently suppressing DC components. A wide common-mode input range (WCMIR) SAR ADC processes the differential signals, ensuring accurate analog-to-digital conversion. The DCM eliminates the need for DC cancelation loops, simplifying the design and reducing power consumption. Implemented in a 0.18 µm CMOS process, the system occupies only 0.30 mm^2^, making it suitable for multi-channel applications. The system achieves over 60 dB DC dynamic range and consumes only 9.6 µW, demonstrating its efficiency for portable devices. The simulation results validate its ability to process PPG signals across various conditions, offering a scalable solution for advanced biomedical sensing platforms.

## 1. Introduction

Photoplethysmography (PPG) sensing is a non-invasive optical technique widely utilized in healthcare and fitness applications. PPG sensors detect blood volume changes in the microvascular bed of tissues by emitting light from LEDs and capturing the reflected or transmitted signal using photodetectors. This method has become increasingly prominent in wearable devices for monitoring heart rate, blood oxygen levels, and vascular health due to its simplicity and cost-effectiveness [1,2].

PPG’s potential applications extend beyond simple monitoring. It is used in diagnosing cardiovascular diseases, estimating blood pressure, and detecting irregularities like atrial fibrillation [3,4]. Moreover, advancements in data algorithms enable enhanced processing of PPG signals for stress analysis and sleep tracking [5]. As wearable and implantable devices continue to evolve, the demand for compact, low-power, and highly accurate PPG readout systems grows significantly [6].

The principle behind PPG involves light absorption and reflection. LEDs emit light that penetrates the skin, and the reflected light, modulated by blood flow, is detected by a photodetector. This detected signal typically consists of a low-frequency AC component, representing pulsatile blood flow, and a high-level DC component, corresponding to static tissue absorption. AC currents typically range between 0.1 and 10 µA, while the DC currents are several orders higher, reaching up to 100 µA. The frequency range for AC signals is generally between 0.5 Hz and 10 Hz, aligning with the heart rate spectrum [2,7,8,9].

In this paper, we propose a novel PPG readout integrated circuit (IC) that incorporates a differential current mirror (DCM) and a wide input range, low-power monotonic successive approximation register (SAR) analog-to-digital converter (ADC). This design aims to enhance energy efficiency and signal fidelity in PPG sensing systems.

The paper is organized as follows: Chapter I introduces the background and objectives. Chapter II reviews prior studies on PPG readout systems. Chapter III presents the proposed PPG readout ICs. Chapter IV discusses the implementation details. and Chapter V concludes the paper.

## 2. Prior Studies

**Figure 1:**
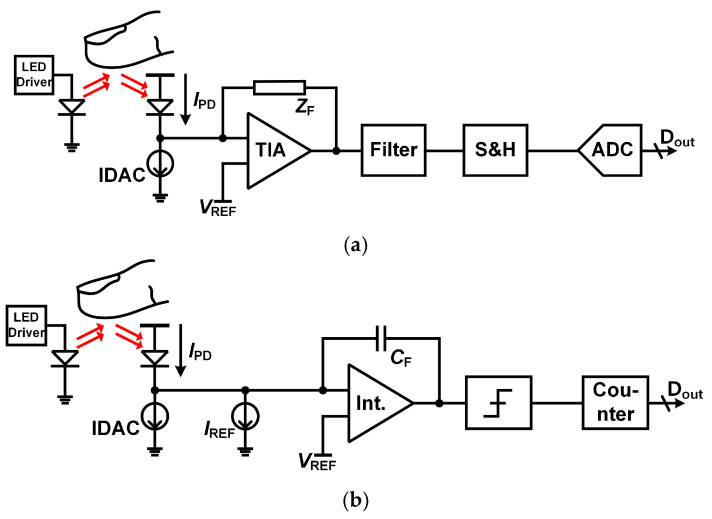
Prior PPG readout system methodologies. (**a**) TIA with ADC, (**b**) LDC.

The development of PPG readout circuits has primarily centered around two architectures: the transimpedance amplifier (TIA) with analog-to-digital conversion (ADC) in a sequential approach and the light-to-digital conversion (LDC) method. These two approaches are widely used due to their specific merits and application relevance, as shown in Figure 1.

### 2.1. TIA with ADC Architecture

As illustrated in Figure 1a, the TIA with ADC method is one of the most used designs. A photodetector receives light signals reflected from the skin, converting them into a current signal. The TIA converts this current signal into a voltage, which is subsequently digitized by the ADC. The key advantage of this method is its modularity, enabling the TIA and ADC to be optimized separately. This architecture effectively addresses noise and enhances signal integrity during the current-to-voltage and voltage-to-digital conversions [10,11,12].

However, this approach has certain limitations. The two-step conversion process can result in higher latency and increased power consumption. Additionally, the TIA design requires careful consideration of bandwidth and noise, particularly for low-amplitude AC signals. Despite these drawbacks, its robustness and ease of integration make it a reliable choice for wearable PPG systems [13,14].

### 2.2. LDC Structures Architecture

The LDC architecture, depicted in Figure 1b, simplifies the readout process by directly converting the current from the photodetector into a digital signal, bypassing the intermediate voltage stage. This design eliminates the need for a TIA, thereby reducing the circuit complexity and power consumption. The key advantage of the LDC method is its high-speed operation due to the single-step conversion process. This makes it particularly suitable for applications demanding rapid data acquisition [15,16].

LDCs are often more susceptible to noise because the direct current-to-digital conversion process lacks intermediate signal conditioning stages. Additionally, this architecture can be challenging to design for wide dynamic ranges without compromising signal fidelity [1,17,18].

### 2.3. Dynamic Range Challenges in Both Architectures

Both architectures share a common feature: the inclusion of auxiliary feedback loops to suppress DC currents. These loops are critical for handling the wide dynamic range of the PPG signals, where the DC current (corresponding to static tissue absorption) can significantly overshadow the AC current (representing pulsatile blood flow). The auxiliary loop ensures that the DC component is suppressed, allowing the AC signal to be accurately captured and processed.

However, this auxiliary loop presents challenges. The DC current levels vary significantly between individuals due to differences in skin tone, tissue composition, and the intensity of reflected light. These variations require the feedback loop to settle before an accurate value can be obtained. This settling process must be repeated for every new measurement, leading to increased response times in mobile PPG devices.

In wearable applications, where user experience demands quick and accurate measurements, this settling time is a notable limitation. Achieving a balance between fast settling and high accuracy remains a critical challenge for both TIA with ADC and LDC architectures. Addressing this issue is essential for the next generation of PPG readout circuits, where efficiency, speed, and accuracy must coexist seamlessly.

## 3. Proposed PPG Readout System

**Figure 2:**
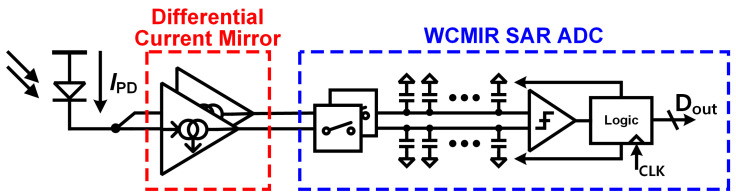
Proposed PPG readout system with DCM and input rail-to-rail SAR ADC.

The proposed PPG readout system employs a differential current mirror (DCM) and a differential SAR ADC architecture, as depicted in Figure 2. The DCM converts the single-ended PPG current signal from the photodetector into a differential voltage signal, which enhances noise immunity and common-mode rejection. The differential SAR ADC then processes the DCM output and automatically suppresses the DC component of the PPG signal, isolating the AC component that contains vital physiological information. The following subsections will provide an in-depth explanation of the key components of the proposed system.

### 3.1. Differential Current Mirror (DCM) for Auto-DC-Suppression

**Figure 3:**
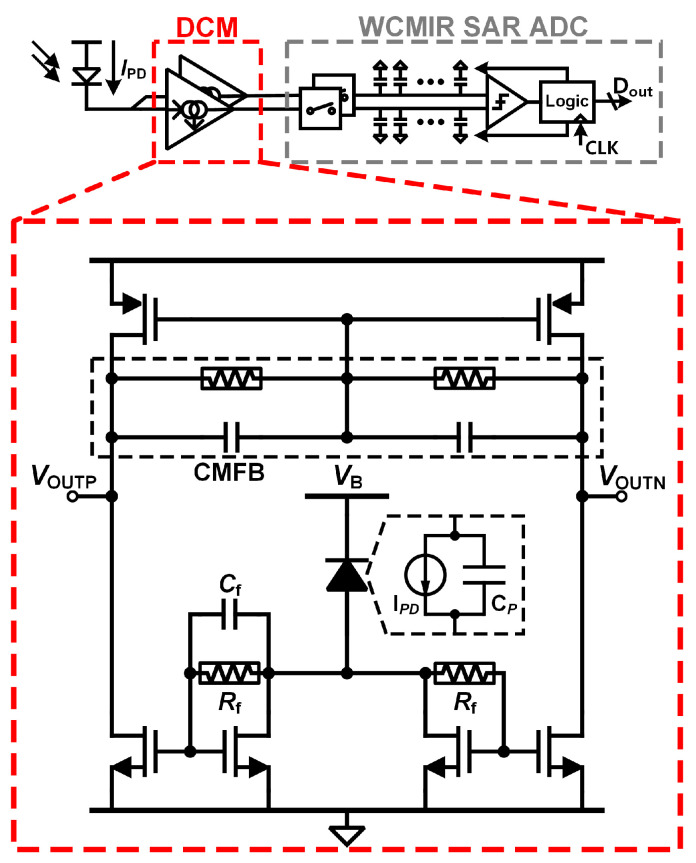
Proposed PPG readout front-end circuits with differential current mirror (DCM).

The DCM employed in this study [19] is distinct from conventional current mirrors, as illustrated in Figure 3. It incorporates two NMOS transistors that are self-biased through a resistor *R_f_*. Uniquely, only one of these transistors has a capacitor *C_f_* connected in parallel with *R_f_*. Considering the low-frequency range of the PPG signal, *R_f_* was implemented using a pseudo-resistor. This design choice ensures extremely high resistance values, which are critical for maintaining appropriate gain and signal integrity at low frequencies while minimizing power consumption. This configuration enables the DCM to split the PPG signal into two distinct signal paths, each with different characteristics.

While previous works [19] utilized a single-ended DCM, the proposed differential topology significantly improves the common-mode rejection ratio (CMRR). However, this structure introduces a potential increase in DC voltage at the output, which we mitigate through the proposed SAR ADC with a wide input common-mode range. This combination ensures robust performance in real-world biomedical applications.

In the signal path where *C_f_* is connected, the high-frequency input impedance is significantly lower compared to the path without *C_f_*. As a result, the AC components of the current generated by the photodetector (PD) predominantly flow through the *C_f_*-connected path. Conversely, the DC component of the PPG current equally flows through both paths, appearing symmetrically across the differential outputs of the DCM. When the differential output is processed by the subsequent ADC, the DC components naturally cancel out, leaving only the AC component for conversion.

This structure offers several notable advantages:

1. No Need for DC Cancelation Loop: Unlike traditional PPG readout ICs, which require an additional DC cancelation feedback loop, this design inherently suppresses the DC component without extra circuitry, simplifying the overall system architecture.
2. Differential Signal Conversion: The ability to convert a single-ended input signal into a differential output enhances noise rejection, particularly against external common-mode interference, which is a significant advantage in wearable and portable devices.

However, the DCM also introduces a design limitation. If the input DC current is smaller than the expected value during design, the output DC voltage of the DCM increases proportionally. This creates a challenge for the subsequent ADC, as it necessitates a wider common-mode input voltage range to accommodate the elevated output voltage of the DCM. Addressing this issue highlights the importance of the wide common-mode input range ADC, which will be discussed in detail in the next subsection.

Although the settling time of the proposed system is relatively long due to the use of pseudo-resistors, the SAR ADC with a wide input range can still quantize the differential signals accurately even before full settling is achieved. This ensures continuous data acquisition without additional power-consuming DC cancelation loops, differentiating our approach from conventional designs.

### 3.2. Wide Common-Mode Input Range (WCMIR) SAR ADC with Input Rail-to-Rail Comparator

In the proposed system, the PPG signal output from DCM is in a differential structure. To efficiently process the differential output signal, a differential ADC, specifically a monotonic SAR ADC [20], is employed. This architecture allows the digitized output to inherently suppress the DC component of the PPG signal, ensuring the preservation of the AC signal that carries vital physiological information. Additionally, the monotonic SAR ADC minimizes switching energy by employing a stepwise capacitor switching scheme, which reduces power consumption significantly compared to conventional SAR ADCs. This low-power feature, combined with its ability to operate effectively over a wide differential input dynamic range, makes it particularly well-suited for wearable and portable biomedical applications where energy efficiency and signal accuracy are paramount.

However, the monotonic SAR ADC has a limitation when dealing with high input common-mode voltages. Due to the use of PMOS transistors in the comparator’s input stage, there is a risk of MSB (Most Significant Bit) decision errors at elevated input common-mode levels. This issue arises because the comparator’s PMOS input stage cannot maintain accuracy under such conditions. Furthermore, if the current generated by the PPG photodetector (PD) is lower than the designed expectation, the common-mode DC voltage at the input of the ADC can increase. This rise in common-mode voltage exacerbates the challenge of accurate conversion, as the comparator’s input transistors may operate outside their optimal range, leading to further decision errors.

**Figure 4:**
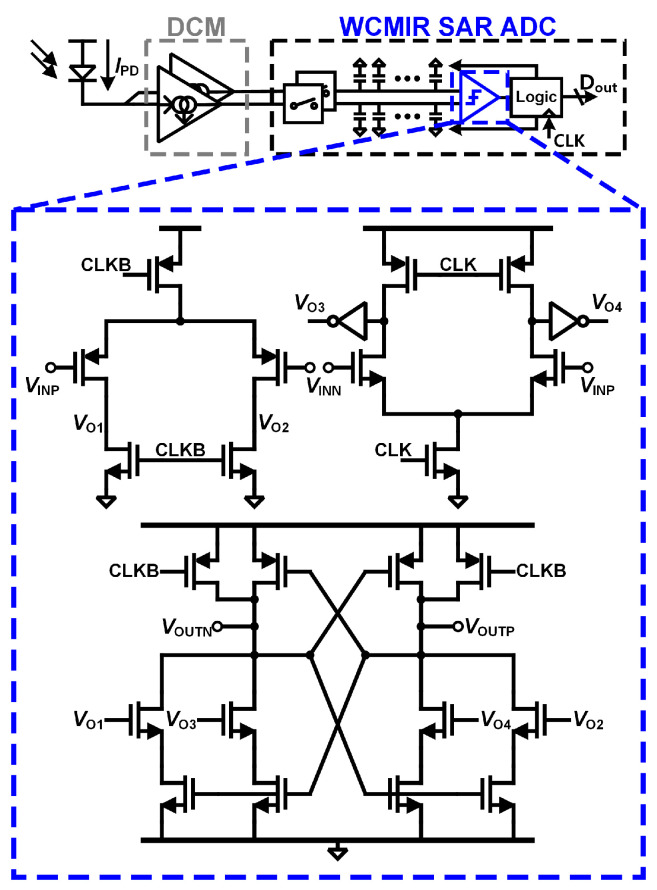
Proposed wide common-mode input range SAR ADC with input rail-to-rail comparator.

To address this challenge, a straightforward solution would be to use a comparator with rail-to-rail input capability. However, in the context of low-power SAR ADCs, it is essential to ensure that such comparators do not generate a shoot-through current during operation. The proposed input rail-to-rail comparator is designed specifically to maintain the low-power characteristics of the SAR ADC, unlike conventional rail-to-rail comparators, which tend to have higher power consumption. The optimization of the comparator structure ensures efficient operation within the power constraints of biomedical readout circuits. To meet these requirements, the proposed wide common-mode input range SAR ADC incorporates a two-stage strong-arm latched comparator [21] with a modified latch stage. Specifically, a parallel structure like NOR gates is introduced into the latch stage (Figure 4), enabling the comparator to maintain accuracy even at high input common-mode voltages.

This design proposal allows NMOS transistors in the comparator’s input stage to handle the MSB decision. As a result, the comparator ensures accurate analog-to-digital conversion, regardless of input common-mode voltage levels. This solution not only resolves the MSB decision issue but also preserves the low-power characteristics essential for wearable and portable PPG monitoring systems.

## 4. Implementation and Simulation Results

In this section, we present the implementation details and simulation results of the proposed PPG readout system. The key components, including the DCM and the WCMIR SAR ADC, are discussed in terms of their design considerations and performance. The simulation results validate the effectiveness of the proposed system in achieving low power consumption, high accuracy, and robust DC suppression. This study is based on the pre-fabrication simulation results; hence, minor discrepancies with actual silicon measurements may exist. However, extensive Monte Carlo and process corner simulations have been conducted to ensure robustness. Future work will involve further optimizations based on fabricated prototype measurements.

### 4.1. Implementation Summary

**Figure 5:**
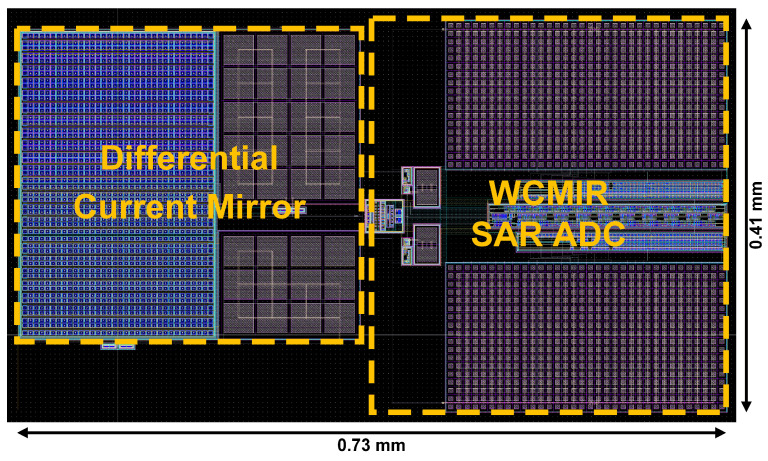
Layout of proposed PPG readout systems.

The proposed PPG system was implemented using a PDK of 0.18 µm CMOS process technology, ensuring compatibility with low-power biomedical applications. As shown in Figure 5 and Table 1 and Table 2, the implemented system occupies 0.30 mm^2^ with only 9.6 μW power consumption, making it compact enough to accommodate multi-channel applications effectively, even as the number of channels increases. The comparison of power consumption values has been reviewed and estimated to ensure a fair and accurate evaluation against previous works. The input-referred noise performance was simulated to be 2.8 pA_rms_ in the condition with 100 nA of *I*_PD_, demonstrating excellent signal fidelity suitable for low-amplitude PPG signals. The proposed WCMIR SAR ADC exhibited a SNDR performance of 60 dB with normal input common voltage, ensuring high enough conversion accuracy. The proposed system achieves a DC Subtraction dynamic range of over 60 dB (60 dB for 100 μA ~ 100 nA), effectively suppressing the DC component while preserving the AC signal and eliminating the need for external DC cancelation loops.

### 4.2. Detailed Simulation Results

**Figure 6:**
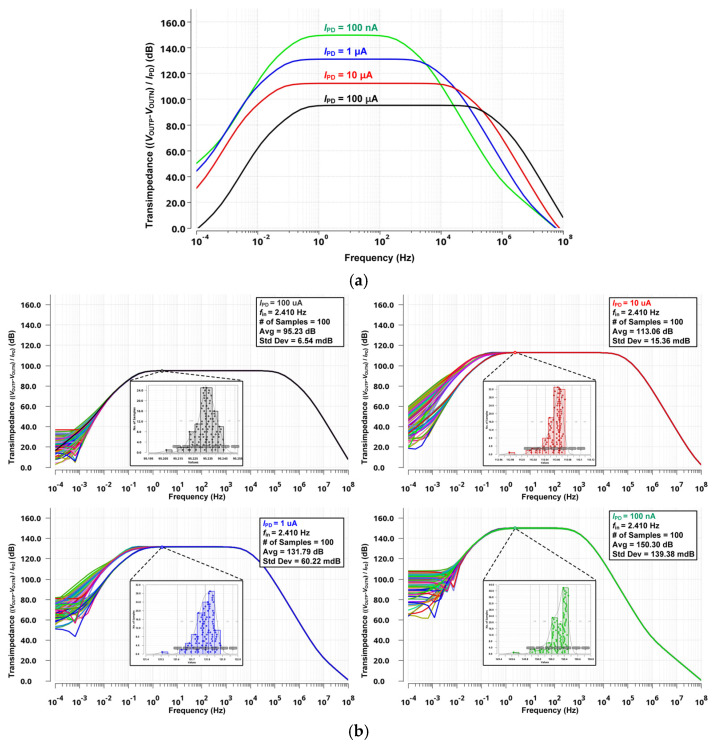

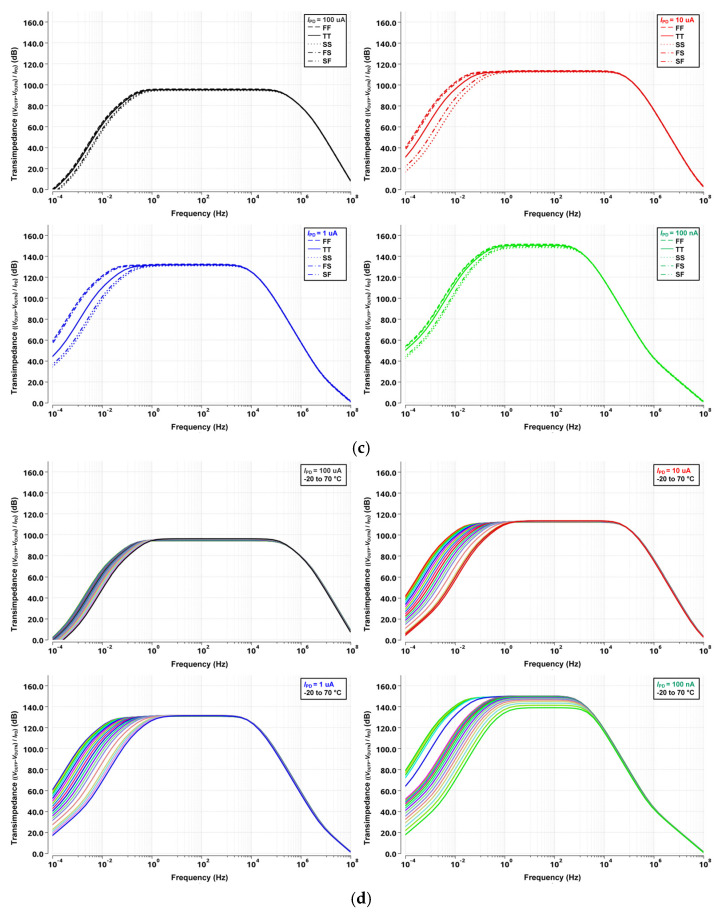
Simulated results of transfer function for DCM in the proposed PPG readout system. (**a**) Transimpedance results in different input current conditions, (**b**) in Monte Carlo variations, (**c**) in different process corners, (**d**) in variable temperature ranges for different current conditions.

Figure 6a illustrates the transfer functions of the proposed DCM for different *I*_PD_ values. To verify the robustness of the proposed design, Monte Carlo simulations were performed, and the results were incorporated into the transimpedance plot in Figure 6b. These results confirm that the circuit maintains stable performance under process variations and device mismatch, ensuring reliable operation across different conditions. In addition to Monte Carlo simulations, process and environmental simulations were conducted to evaluate the robustness of the proposed readout system under corner and temperature variations, as shown in Figure 6c,d. These results further confirm the stable operation and performance reliability of the system under real-world conditions. The results confirm that the DCM maintains a sufficiently high and stable transconductance across the bandwidth of the PPG input signal, ensuring reliable signal amplification. As the *I*_PD_ value decreases, an increase in transconductance is observed. This behavior occurs because the output impedance of the DCM increases at lower input current levels, leading to a corresponding rise in transconductance. The ability to maintain stable transconductance within the PPG signal bandwidth highlights the effectiveness of the proposed DCM design in handling varying photodetector currents while preserving signal integrity.

**Figure 7:**
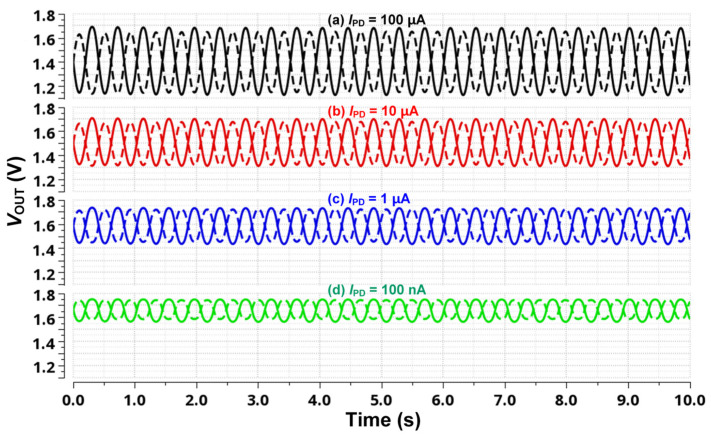
Simulated results of transient response for DCM in the proposed PPG readout system with different DC current situations.

Figure 7 presents the transient response of the proposed DCM under varying *I*_PD_ conditions. The simulation results illustrate how the DCM dynamically responds to changes in the *I*_PD_, maintaining proper signal conversion in typical operating conditions. However, when the *I*_PD_ decreases to an extremely low level, the DC output level of the DCM rises significantly, reaching up to 1.7 V. This excessive shift in DC level can pose a challenge for conventional differential SAR ADCs, which typically have a limited common-mode input range. These results clearly demonstrate the necessity of the WCMIR SAR ADC, which ensures accurate analog-to-digital conversion even when the input common-mode voltage varies significantly. By incorporating the proposed SAR ADC, the system effectively mitigates the limitations of standard ADC designs, enabling stable and precise PPG signal processing across a broad range of operating conditions.

**Figure 8:**
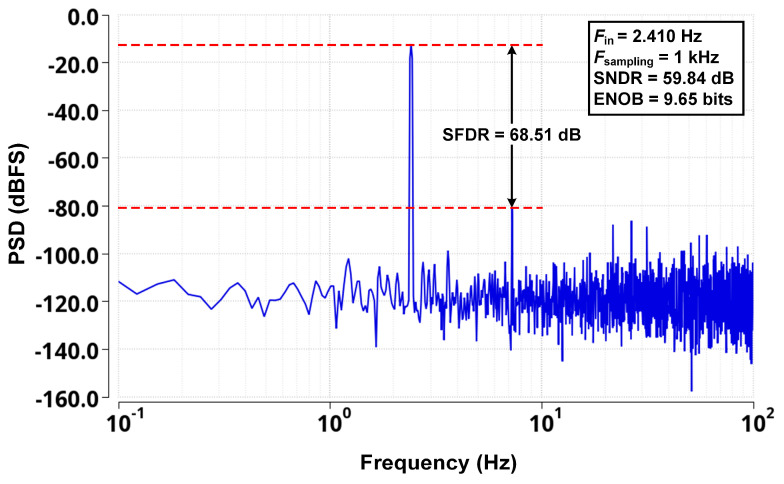
Simulated FFT spectrum of the proposed WCMIR SAR ADC.

Figure 8 illustrates the full-scale sine wave test results of the proposed WCMIR SAR ADC. The results confirm that the ADC maintains stable and accurate signal conversion across the entire input range.

**Figure 9:**
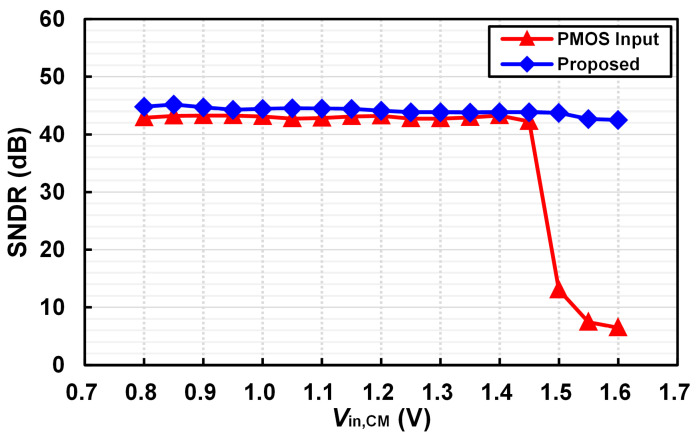
Compared SNDR at *V*_in_ = 0.8 V_pp_ between the conventional and proposed SAR ADC.

Figure 9 compares the SNDR of the conventional monotonic SAR ADC [20] and the proposed WCMIR SAR ADC as a function of the input common-mode voltage. The results clearly illustrate that when the common-mode voltage exceeds 1.4–1.5 V, the conventional monotonic SAR ADC exhibits severe performance degradation, producing unreliable test results. This is attributed to the comparator’s PMOS input stage failing to maintain accurate MSB decisions under high common-mode voltages. In contrast, the proposed WCMIR SAR ADC maintains stable SNDR performance across the entire input common-mode voltage range, demonstrating its robustness against variations in common-mode levels. The enhanced comparator design with NOR-gate-based latching ensures accurate ADC operation even at high common-mode voltages, validating the necessity and effectiveness of the proposed ADC architecture for low-power biomedical applications.

## 5. Conclusions

In this work, a low-power PPG readout IC was proposed, utilizing DCM and WCMIR SAR ADC to achieve automatic DC suppression and robust differential signal processing. The architecture eliminates the need for traditional DC cancelation loops and demonstrates excellent performance in terms of power efficiency, noise immunity, and dynamic range. Simulations confirmed their suitability for wearable and portable biomedical applications.

Future work could explore further optimization of the SAR ADC for higher resolution and integration with advanced signal processing techniques, such as machine learning, to enable real-time health monitoring. The proposed design also lays the groundwork for integrating other biopotentials, such as ECG or SpO_2_, into a unified sensing platform for comprehensive health diagnostics.

## Figures and Tables

**Table 1:** Performance comparisons.

	[10] JSSC’17	[11] JSSC’18	[16] TBCAS’20	[17] SSC-L’20	This Work
Technology	0.13 mm	0.18 mm	0.18 mm	0.18 mm	0.18 mm
Area	-	1.75 mm^2^	2.5 mm^2^	1.25 mm^2^	0.30 mm^2^
Power Supply	1.2/1.5/3.3 V	1.2/3.3 V	1.2/3.3 V	1/2.5 V	1.8 V
RX Power Consumption	50 mW	89 mW	61 / 89 mW	8.1 mW	9.6 mW (DCM) 0.11 uW (ADC)
Max. Input Referred Noise	0.034 nA_rms_	N/A	5.7 pA/√Hz	N/A	2.8 pA_rms_ @ *I*_PD_ = 100 nA
DR of DC Subtraction	*I*_max_ = 12 mA	7.6 Bit (*I*_max_ = 10 mA, *I*_min_ = 20 nA)	7 Bit (*I*_min_ = 20 nA ~ 2 mA)	8 Bit (*I*_max_ = 56.3 mA)	60 dB (*I*_max_ = 100 mA, *I*_min_ = 100 nA)
DC Suppression Loop	O	O	O	O	X
SNDR of ADC	N/A	N/A	N/A	N/A	59.8 dB
Common-Mode Input Range of ADC	N/A	N/A	N/A	N/A	~1.8 V
Architecture	TIA + ADC	TIA + ADC	LDC	LDC	DCM + ADC

**Table 2:** Input-referred noise.

*I*_PD_	Input-Referred Noise
100 μA	5.185 nA_rms_
10 μA	237.6 pA_rms_
1 μA	26.6 pA_rms_
100 nA	2.8 pA_rms_

## Data Availability

The original contributions presented in this study are included in the article. Further inquiries can be directed to the corresponding author.

## References

[1] Asada H.H., Shaltis P., Reisner A., Rhee S., Hutchinson R.C. (2003). Mobile monitoring with wearable photoplethysmographic biosensors. IEEE Eng. Med. Biol. Mag..

[2] Ebrahimi Z., Gosselin B. (2023). Ultralow-Power Photoplethysmography (PPG) Sensors: A Methodological Review. IEEE Sens. J..

[3] Webster J.G. (1997). Design of Pulse Oximeters.

[4] Shin H., Min S.D. (2017). Feasibility study for the non-invasive blood pressure estimation based on PPG morphology: Normotensive subject study. Biomed. Eng. Online.

[5] Kim D.H., Lee E., Kim J., Park P., Cho S. (2020). A Sleep Apnea Monitoring IC for Respiration, Heart-Rate, SpO_2_ and Pulse-Transit Time Measurement Using Thermistor, PPG and Body-Channel Communication. IEEE Sens. J..

[6] Charlton P.H., Kyriacou P.A., Mant J., Marozas V., Chowienczyk P., Alastruey J. (2022). Wearable photoplethysmography for cardiovascular monitoring. Proc. IEEE.

[7] Kyriacou P.A., Allen J. (2021). Photoplethysmography: Technology, Signal Analysis and Applications.

[8] Glaros K.N., Drakakis E.M. (2013). A Sub-mW Fully-Integrated Pulse Oximeter Front-End. IEEE Trans. Biomed. Circuits Syst..

[9] Chettri N., Aprile A., Bonizzoni E., Malcovati P. (2024). Advances in PPG Sensors Data Acquisition with Light-to-Digital Converters: A Review. IEEE Sens. J..

[10] Sharma A., Polley A., Lee S.B., Narayanan S., Li W., Sculley T., Ramaswamy S. (2017). A Sub-60-μA Multimodal Smart Biosensing SoC with >80-dB SNR, 35-μA Photoplethysmography Signal Chain. IEEE J. Solid State Circuits.

[11] Schönle P., Glaser F., Burger T., Rovere G., Benini L., Huang Q. (2018). A Multi-Sensor and Parallel Processing SoC for Miniaturized Medical Instrumentation. IEEE J. Solid State Circuits.

[12] Caizzone A., Boukhayma A., Enz C. (2017). Comprehensive Noise Analysis in PPG Read-Out Chains. Proceedings of the International Conference on Noise and Fluctuations (ICNF).

[13] Xu J., Konijnenburg M., Song S., Ha H., van Wegberg R., Mazzillo M., Fallica G., Van Hoof C., De Raedt W., Van Helleputte N. (2018). A 665 μW Silicon Photomultiplier-Based NIRS/EEG/EIT Monitoring ASIC for Wearable Functional Brain Imaging. IEEE Trans. Biomed. Circuits Syst..

[14] Konijnenburg M., van Wegberg R., Song S., Ha H., Sijbers W., Xu J., Stanzione S., van Liempd C., Biswas D., Breeschoten A. (2019). 22.1 A 769μW Battery-Powered Single-Chip SoC with BLE for Multi-Modal Vital Sign Health Patches. Proceedings of the IEEE International Solid-State Circuits Conference (ISSCC), San Francisco, CA, USA, 17–21 February 2019.

[15] Song S., Lin Q., van Hoof C., van Helleputte N. (2020). A 50μW Fully Differential Interface Amplifier with a Current Steering Class AB Output Stage for PPG and NIRS Recordings. IEEE Trans. Circuits Syst. II Exp. Briefs.

[16] Lin Q., Xu J., Song S., Breeschoten A., Konijnenburg M., Van Hoof C., Tavernier F., Van Helleputte N. (2020). A 119 dB Dynamic Range Charge Counting Light-to-Digital Converter for Wearable PPG/NIRS Monitoring Applications. IEEE Trans. Biomed. Circuits Syst..

[17] Marefat F., Erfani R., Mohseni P. (2020). A 1-V 8.1-μW PPG-Recording Front-End with >92-dB DR Using Light-to-Digital Conversion with Signal-Aware DC Subtraction and Ambient Light Removal. IEEE Solid State Circuits Lett..

[18] Marefat F., Erfani R., Kilgore K.L., Mohseni P. (2020). A 280 μW 108 dB DR Readout IC with Wireless Capacitive Powering Using a Dual-Output Regulating Rectifier for Implantable PPG Recording. IEEE Int. Solid State Circuits Conf. (ISSCC) Dig. Tech. Papers.

[19] Noh H., Kim W., Jeon H. (2024). A Photodiode Sensor Readout Circuit Utilizing a Differential Current Mirror for Dark Current Cancellation. J. Integr. Circuits Syst..

[20] Liu C.-C., Chang S.-J., Huang G.-Y., Lin Y.-Z. (2010). A 10-bit 50-MS/s SAR ADC with a monotonic capacitor switching procedure. IEEE J. Solid State Circuits.

[21] van Elzakker M., van Tuijl E., Geraedts P., Schinkel D., Klumperink E.A.M., Nauta B. (2010). A 10-bit Charge-Redistribution ADC Consuming 1.9 μW at 1 MS/s. IEEE J. Solid State Circuits.

